# *Salmonella enterica *changes *Macrosteles quadrilineatus *feeding behaviors resulting in altered *S. enterica* distribution on leaves and increased populations

**DOI:** 10.1038/s41598-022-11750-3

**Published:** 2022-05-20

**Authors:** Victoria L. Harrod, Russell L. Groves, Ellie G. Guillemette, Jeri D. Barak

**Affiliations:** 1 Department of Entomology, University of Wisconsin-Madison, Madison, Wisconsin USA; 2 Department of Plant Pathology, University of Wisconsin-Madison, Madison, Wisconsin USA

**Keywords:** Microbiology, Plant sciences

## Abstract

Hemipteran insects are ubiquitous inhabitants of the phyllosphere. Changes in microbial phyllosphere communities have recently been demonstrated following infestation by *Macrosteles quadrilineatus* (Aster Leafhopper). Although epiphytic *Salmonella enterica* populations naturally decline in the phyllosphere of plants, *M. quadrilineatus* infestation facilitated the growth of the bacterial pathogen populations. Here, we demonstrate that cellular damage by insect stylet penetration results in a localized beneficial niche on the leaf surface, leading to enhanced *S. enterica* populations. We measured *S. enterica* populations and colonization patterns on plants infested with Hemipterans with distinct feeding behaviors. *M. quadrilineatus* infestation resulted in higher solute leakage and significantly greater bacterial populations than plants absent of insects. Following immigration via contaminated irrigation water, the highest populations of *S. enterica* are naturally found on the tips of tomato leaflets. We discovered *M. quadrilineatus* feeding preference altered the natural distribution of *S. enterica* populations, and that the presence of *S. enterica* altered the distribution of probing attempts. These findings elucidate how cellular damage resulting from insect feeding drives changes in bacterial colonization of the phyllosphere.

## Introduction

*Salmonella enterica*, a human enteric bacterial pathogen, has recently been recognized as a member of the phyllosphere microbiome^1,2^. Unlike most members of this microbiome, the presence of *S. enterica* annually leads to food borne illness from the consumption of fresh, raw produce. In fact, cases of salmonellosis derived from the consumption of contaminated fresh produce has steadily increased over the last decade^3^. *S. enterica* colonization of the phyllosphere is reported to begin with contaminated irrigation water or direct application of raw (vs. composted) manure as a soil amendment^4–6^. Irrigation water is not only a conduit for *S. enterica* contamination directly to the phyllosphere, but can further spread the human pathogen from plant to plant via splash dispersal^7^. Here again, application of raw manure has been implicated as an avenue for contamination of agricultural fields but also has been reported to stimulate enteric bacterial growth on subsequent crops^1^. These human-mediated practices, among many others, aid *S. enterica* in achieving access to preharvest produce, thereby generating a scenario that may lead to foodborne illness.

While pathogen introduction to leaves preharvest is the first step in a sequence culminating in human illness, *S. enterica* populations have been observed to decline in the phyllosphere of healthy plants^8–10^. Hostile environmental conditions, such as direct UV radiation, desiccation, and a lack of nutrient availability, are a few of the limiting factors prompting bacterial populations to decrease over time**.** The high proportion of *S. enterica* outbreaks associated with consumption of fresh, raw produce, however, indicate that these bacteria have evolved to exploit several biological niches to successfully persist. Following contamination of the phyllosphere via irrigation water, *S. enterica* populations concentrate around glandular trichomes and stomates, two ubiquitous leaf structures that exude scarce metabolites or provide leaf internalization access respectively, each resulting in a beneficial niche for epiphytic bacteria^11^. Furthermore, *S. enterica* successfully persists near leaf regions altered by phytobacterial pathogens, such as *Xanthomonas* species^12^. These authors hypothesize that *S. enterica* uses nutrients made available during the plant infection process.

Just as a subset of phytobacterial pathogens were discovered as potential biological multipliers, we previously identified phytophagous insects as additional promoters for *S. enterica* survival in the phyllosphere^13^. Specifically, our lab uncovered that *Macrosteles quadrilineatus* (Aster leafhopper) infestation significantly promoted *S. enterica* populations and persistence overtime on both lettuce and tomato leaves^9,13^. Although this relationship was previously discovered, the mechanisms by which the insect facilitates the persistence of these bacterial populations has not yet been established.

We hypothesize that insect feeding alters the phyllosphere from a *S. enterica* inhospitable habitat to an inhabitable niche. Members of the Hemipteran order of insects utilize a narrow and segmented piercing-sucking mouthpart, collectively composed of stylets, to feed on the phloem or xylem of plants. Although Hemipterans collectively share these mouthparts, different members of this important group employ unique probing and feeding strategies that elicit distinctive plant responses. For instance, the stylet of an aphid (Hemiptera, Stenorrhyncha, Aphidoidea) reaches the phloem via an intercellular pathway. After puncturing the epidermis, an aphid’s stylet transiently probes and injects adjacent cells with watery saliva^14,15^ prompting upregulation of the salicylic acid pathway^16,17^. Contrastingly, leafhoppers (Hemiptera, Auchenorrhyncha, Cicadellidae) feed intracellularly by sieving through layers of cells to reach into the phloem, consequently upregulating the jasmonic acid pathway^18^. To date, the extent of cellular damage, elicited by feeding, has not been measured for Hemipterans. We hypothesize that these two modes of stylet penetration cause varying levels of cellular damage, and thus may uniquely alter the infested phyllosphere.

Here, we explore how differences between *M. quadrilineatus* and *M. persicae* feeding behaviors could influence the extent of cellular damage, and further how these differences may alter the phyllosphere for subsequent bacterial populations. We use *S. enterica* as a biological reporter of changes to the phyllosphere resulting from insect feeding, and lettuce and tomato plants were utilized as relevant plant systems for our experiments given their repeated association with domestic outbreaks of salmonellosis^19,20^. We hypothesize that preferred feeding locations of insects will experience higher levels of cellular damage, and in turn be associated with enhanced *S. enterica* populations^21^. To explore this hypothesis, we mapped preferential *S. enterica* colonization sites, preferred feeding locations of *M. quadrilineatus*, and examined whether earlier insect infestation influenced these distributions. In turn, we also investigated whether leaves previously contaminated with *S. enterica* would influence the feeding biology of *M. quadrilineatus* or *M. persicae* in the phyllosphere. Results from this study illustrate how unique hemipteran feeding behaviors can alter the phyllosphere and subsequent microbial community, with special reference to *S. enterica*.

## Materials and methods

### Bacterial strains, media, and culture conditions

A kanamycin (Kan) resistant strain of *S. enterica* serovar Typhimurium 14028 s, from -80 °C freezer stocks, were utilized and grown in a lysogeny broth (LB; Difco LB Broth) at 37 °C, shaking overnight at 200 rpm. *S. enterica* cultures were normalized to an optical density at 600 nm of 0.2 in sterile water*.* Inoculum preparations were verified by enumerating populations following serial dilution, plating on Kan amended plates, and incubated overnight at 37 °C.

### Insect rearing

Colonies of *Macrosteles quadrilineatus* were maintained on oat seedlings (*Avena sativa*) under a constant temperature of 27 °C and a 16:8 (L:D) photoperiod. A colony of *Myzus persicae* was provided by Jason Timothy Ingram and Dr. Stewart Gray (Cornell University) and maintained on turnip plants (*Brassica rapa*) under the same controlled conditions of 27 °C and a 16:8 (L:D) photoperiod. Voucher specimens of adult female and male *M. quadrilineatus* and apterous *M. persicae* from our colony were deposited in the Wisconsin Insect Research Collection, University of Wisconsin (http://labs.russell.wisc.edu/wirc/).

### Plant assays

*Solanum lycopersicum* (tomato, cv. Money Maker), and *Lactuca sativa* (lettuce, cv. Butterhead) seedlings were cultivated using Professional Growing Mix (Sunshine Redi-earth) in 6″ pots held in a growth room maintained at a 16:8 (L:D) photoperiod and 24 °C light and 19 °C dark conditions. No plant material was collected. Seeds were bought commercially (Eden Brothers). Tomato plants were established and maintained for five weeks prior to all experiments, whereas lettuce plants were grown and utilized after six weeks. Six sets of 4.5 cm diameter plexiglass clip cages, fashioned with insect-proof mesh at one end, were fastened with clips onto the abaxial (under) surface of two opposing leaves, three individually containing one adult *M. quadrilineatus* and the remaining three left empty as a control. Each clip cage was attached to the center of leaflets on each plant. Plants were held at a constant 24 °C temperature with a 16:8 (L:D) photoperiod and were randomly assigned treatment groups indicating the length of infestation. At each infestation period of 24, 48, or 72 h, individual *M. quadrilineatus* were removed, and leaf discs were excised from under each clip cage to assess for electrolyte leakage. Similar experiments were carried out with apterous *M. persicae* (single insect per cage) on tomato plants. An additional experiment evaluated whether electrical conductivity measurements differ on tomato leaflets infested with singular or multiple aphids (3 individuals). Before infesting plants, individual *M. quadrilineatus* were collected with a respirator whereas a wet brush was used to transfer *M. persicae*. After the initial collection, insects were placed into a container over ice to impede movement thereby facilitating the transfer into a clip cage. Insects were visually monitored for any movement immediately after plant application to ensure they were not injured during placement.

### Cellular damage

To analyze the extent of cellular damage associated with insect probing and feeding, estimates of electrolyte leakage were obtained by measuring electrical conductivity as previously described^22^. Briefly, a set of three comparable 10 mm-diameter leaf discs from under clip cages with or without insects were placed in a single well of a 12-well tissue culture plate containing 4 ml of sterile water. Plates were positioned on a rotating table at 50 rpm for approximately 30 min, acting as a wash step. This wash step prevented any leaf contaminants, such as remnant soil, from affecting conductivity measurements. Water from each well was subsequently removed and replaced with fresh, sterile water, and electrical conductance was immediately measured. Electrical conductance was measured by pipetting 1 ml of the aqueous solution from sample wells onto an ECTestr11 + MultiRange electrical conductance probe to assess the extent of conductive electrolyte leakage, here used as a proxy for cellular damage. After the initial assessment of electrical conductance, sample plates were left on a lit bench at ambient temperature (24 °C) for 6 h, after which a second and final conductivity measurement was taken. Differences in measured conductance between the two estimates were used for data analysis comparing each treatment group and used as a proxy for electrolyte leakage.

### Distribution of *Salmonella enterica* on the leaf phyllosphere

To characterize the distribution of *S. enterica* on tomato and lettuce plants, attached leaflets and whole leaves, respectively were dip inoculated in a suspension of *S. enterica*. Replicate sets of tomato and lettuce plants were dip-inoculated for one minute in 450 ml of sterile water with the addition of 75 µL of Sil-Wet, or a 10^8^ CFU/ml suspension of *S. enterica* prepared as described above with the addition of 75 µL of Sil-Wet. In each replicate, tip, middle and basal regions of whole leaves (lettuce) and leaflets (tomato) were randomized in a 2X2 factorial design, to receive either *S. enterica* suspensions or water controls. One-hour post-dip inoculation, clip cages were placed onto tip, middle and basal sections of leaflets or leaves for later assessments of electrolyte leakage and *S. enterica* population enumeration. Water and *S. enterica* dip-inoculated plants were then placed in clear, plastic bins held at 24* °C* temperature under a 16:8 (L:D) photoperiod and sampled seventy-two hours post-inoculation. In a complementary experiment designed to evaluate the influence of leaf angle on *S. enterica* distribution and electrolyte leakage *S. enterica* dip-inoculated plants were placed into a modified container with plastic ramps positioning tomato leaves at a 65*°* vertical angle propping the tips of leaflets upwards and above the basal portions of leaves (e.g. petiole attachment). Prior to leaf excision for the two aforementioned experiments, each location (on the tip, middle and basal portions) was assigned a number and was entered into a random group generator, to prescribe the areas which would be used to measure *S. enterica* populations, and associated electrolyte leakage *(*https://www.randomizer.org).

To assess *S. enterica* populations, plants were sampled seventy-two hours after dip-inoculation. Specifically, one 10 mm diameter leaf disc was excised from under clip cages on either lettuce or tomato. Samples were individually homogenized in 500 μl of sterile water using a cordless Dremel tool, and further diluted 1:10 in sterile water. Homogenates were immediately plated on LB-Kan, incubated overnight at 37* °C*, and populations were enumerated after 24 h. Electrolyte leakage was assessed three days after dip-inoculation as previously described. A total of 3 experimental replicates were completed for each experiment.

### *S. enterica*, plant, and insect interaction

An additional experiment was performed to determine if the presence of *M. quadrilineatus* or *M. precise* altered the natural distribution of *S. enterica* populations or the magnitude of electrolyte leakage on tomato leaves. Groups of tomato plants were randomly assigned to treatment groups (water, or *S. enterica)* and arranged as a randomized complete block. One-hour post dip-inoculation, one clear hinged lid container (8 × 5¾ × 3; Dart Container Corporation) was fastened onto a middle-aged leaflet. Clamshell containers were concurrently infested by five, adult *M. quadrilineatus,* or five apterous *M. persicae*, which were allowed to move freely around the entire leaflet, whereas a replicate set of clamshells remained empty for uninfested controls. Replicate sets of 10 mm diameter leaf discs were collected at the tip, middle and basal leaflet portions at 72 h post-infestation and were randomly selected for assessments of *S. enterica* populations or electrolyte leakage (e.g. cellular damage) utilizing a random group generator (https://randomizer.org). A total of 3 experimental replicates were completed.

To determine whether *S. enterica* could influence the feeding behavior of *M. quadrilineatus*, the distribution of salivary sheathes was observed on *S. enterica-*contaminated tomato leaves. Groups of four tomato plants were randomly assigned to the following inoculation groups: whole leaf water inoculation, whole leaf *S. enterica* inoculation, or *S. enterica* inoculated onto basal, middle or tip portions of select leaflets, and organized as a randomized complete block design with 3 experimental replicates. Regions uncontaminated by *S. enterica* were inoculated with sterile water. One hour post dip-inoculation, sets of 5 adult *M. quadrilineatus* were released into experimental clamshells and allowed access to whole leaves with different inoculation treatments. Following 72 h of infestation, all insects were removed and whole leaflets were extracted, stained and cleared to enumerate salivary sheathes.

### Salivary sheath staining and clearing procedure

To enumerate salivary sheaths associated with adult *M. quadrilineatus* feeding, experimental leaflets were extracted, and subsequently stained with 0.2% acid fuchsin in a 1:1 (vol/vol) solution of 85% glacial acetic acid and 95% ethanol, otherwise known as McBryde’s acid fuchsin stain^23,24^. Leaflets were fully submerged within the dye for 20 to 24 h at ambient temperature (24 °C). To remove chlorophyll and clear tissues, leaflets were soaked in 95% ethanol for 30 min, replacing the stained liquid with new ethanol every 10 min to ensure residual dye is washed off. Leaflets were then heated in a 1:1:1 (vol/vol/vol) solution in glycerol, 85% glacial acetic acid, and water, and individually boiled for 8 to 10 min to appear translucent. Salivary sheathes of individual leaflets were visually quantified under an Olympus SZ60 Stereoscope with a white background to better highlight embedded salivary sheathes.

### Feeding and resting preference of *M. quadrilineatus* and *M. persicae *on contaminated plants

To determine the response of adult *M. quadrilineatus’* to leaf surfaces contaminated with *S. enterica*, two observational experiments were performed. In a first set of experiments, one middle-aged leaflet was entirely inoculated with sterile water, *S. enterica,* or both treatments on separate ends (tip or basal end) of leaves. One-hour post-inoculation, a modified clam shell container was affixed to encase each experimental treatment. Each cage was placed on a container at a height that would mimic the natural position of the leaflet and adjusted to ensure that the leaf did not touch the sides of the cage while still attached to the plant. Sets of five adult *M. quadrilineatus* per cage were released and allowed to move freely inside. Approximately 15 min post-infestation, a visual observation was made to assess the location (container, *S. enterica-* or water-inoculated regions) of individual leafhoppers while also noting the position of insects on either abaxial or adaxial leaf surfaces. A total of 8 different visual assessments over 2 h were conducted for each set of leafhoppers, leading to 16 observations per treatment group for one experimental replicate. A total of 5 experimental replicates were completed.

To further define whether *S. enterica* influenced *M. quadrilineatus* and *M. persicae* resting preferences, observations of adult insects were made in terms of their positions across leaflets or the experimental cage. Observations (basal, middle or tip) for sets of *M. quadrilineatus* (5 per plant) were recorded at 2-, 24-, and 48 h post infestation on tomato leaflets inoculated exclusively at the base, middle or tip, or entirely inoculated with *S. enterica* or water. Observations (*S. enterica* or water) for sets of *M. persicae* (1 per clip cage) were recorded at 24-, 48- and 72 h post infestation on *S. enterica* or water inoculated halves of leaflets (tip or basal end). The location of *S. enterica* inoculations were randomly assigned to leaf areas by utilizing a random group generator (https://randomizer.org). Differences in observed times and the alternative inoculation style between insect species was chosen to accommodate the smaller and lesser mobile apterous life stage of *M. persicae*, compared to the larger bodied and more mobile *M. quadrilineatus.* Groups of 4 plants were utilized for each treatment group with 3 experimental replicates. At the conclusion of these experiments, leaflets from *M. quadrilineatus* infested plants were removed and stained in an effort to count salivary sheathes 72 h following insect exposure, as mentioned above.

### Statistical analysis

Student’s t-tests were performed to compare estimates of electrical conductivity of leaflet samples that were uninfested or infested between the two experimental taxa (*M. quadrilineatus* and *M. persicae*), for water inoculated treatments at 24, 48, or 72 h. A one-way, analysis of variance (ANOVA) was used to assess if *S. enterica* populations or electrical conductance measurements varied among regions on leaves/leaflets (notably the basal, middle, or tip regions) of uninfested tomato or lettuce plants in their natural position, tomato leaflets altered by a 65° upward-angled ramp, and tomato plants that were infested by either *M. quadrilineatus* or *M. persicae*. Furthermore, ANOVA was used to determine the distribution of *M. quadrilineatus* salivary sheathes across tomato leaflets uniquely inoculated at the base, middle or basal regions, or remained entirely inoculated with either sterile water or *S. enterica*. Interpolation was used to visualize estimated *S. enterica* populations outside of the pre-determined leaf excision points (tip, middle and basal regions) using the ‘lattice’ and ‘akima’ packages on R-Studio. An analysis of covariance (ANCOVA) was used to analyze the proportion of resting *M. quadrilineatus* and *M. persicae* across the experimental cage and tomato leaflets with half-inoculation of *S. enterica* and water on opposing leaflet ends. *M. quadrilineatus* resting preference for uniquely *S. enterica* inoculated surfaces (tip, middle and basal regions), or alternative surfaces (water, or experimental cage), was determined using a likelihood ratio chi-square test.

## Results

### *M. quadrilineatus* infestation (intracellular penetration) results in greater cellular damage than uninfested plants

**Figure 1:**
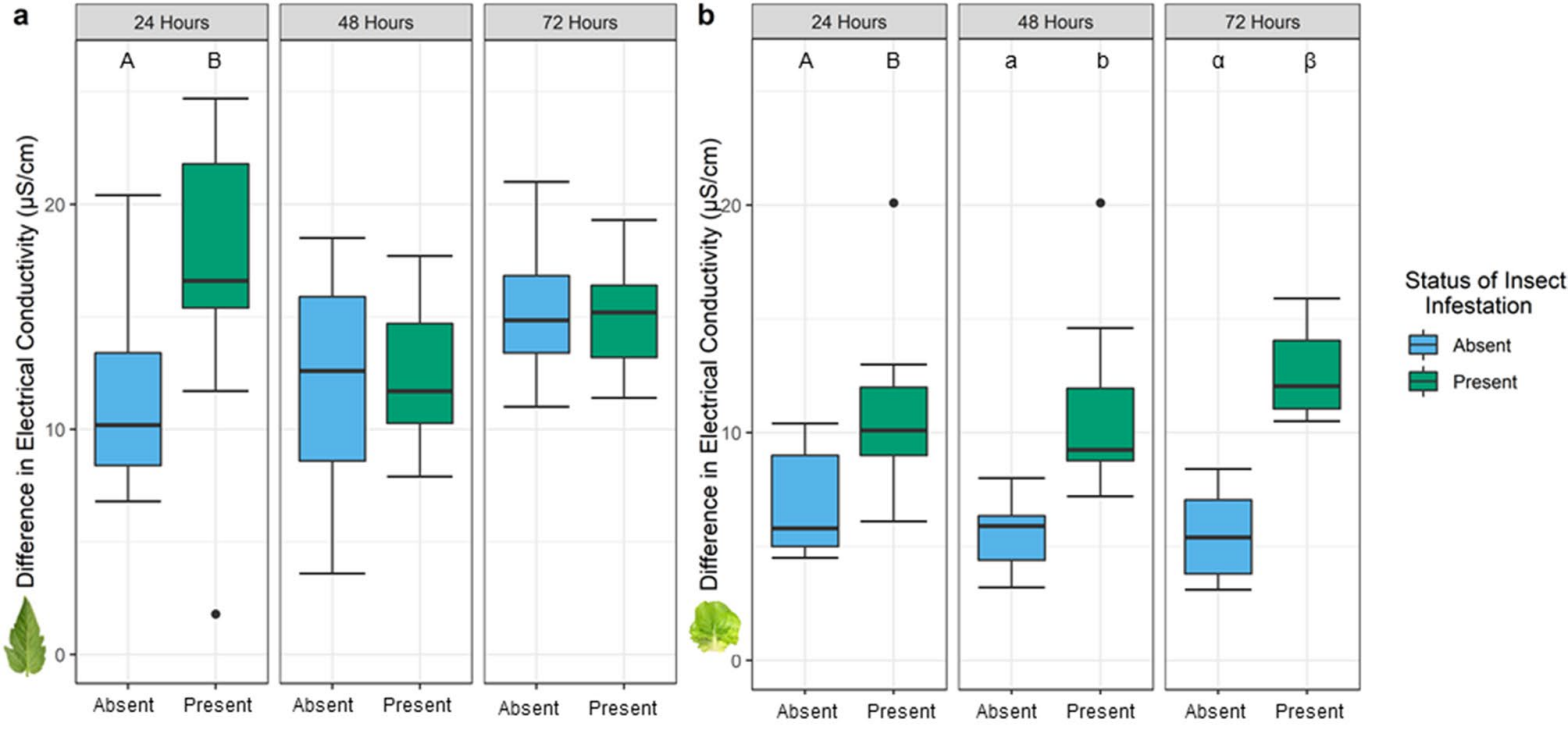
Infestation by *Macrosteles quadrilineatus* leads to an increase in measured cellular damage on tomato (**a**) and lettuce (**b**) leaves. When compared to uninfested areas, electrolyte leakage was significantly higher on tomato leaflets (**a**) infested for 24 h, and on lettuce leaves (**b**) infested for 24, 48, and 72 h (*P* < 0.05). Three clip cages were fastened onto a middle leaf each containing one leafhopper (green), and additional three clip cages remained empty (blue) on an opposing leaf. Electrical conductance was calculated by subtracting the final from the initial measurement for damaged and undamaged leaf discs and were used to evaluate the extent of electrolyte leakage over six hours. Letters above boxplot indicate significant differences between treatment groups within a single time point (*P* < 0.05). A student’s t-test was used to assess significance between samples from infested or non-infested clip cages. Singular dots represent an outlier point.

To further investigate how different feeding styles alter the phyllosphere, we analyzed the extent of electrolyte leakage (using electrical conductivity as a proxy) on tomato plants in response to intracellular or intercellular penetration employed by leafhoppers and aphids, respectively. Tomato plants infested with leafhoppers had significantly higher levels of measured electrical conductivity at 24 h post-infestation (hpi) when compared to plants with no insects (*P* < 0.0005; Fig. 1a). After 24 hpi, measurements of electrical conductivity were not significantly different on tomato plants infested by *M. quadrilineatus*, or without insects (*P* > 0.05; Fig. 1a). In the presence of aphids, measurements of electrical conductivity were not significantly different between plants with or without insects at the measurement timepoints of 24, 48, or 72 hpi (*P* > 0.05; Supplemental Fig. S1). A complimentary experiment determined that increasing aphid populations, from one individual to three, did not influence the extent of electrolyte leakage on tomato plants (*P* = 0.398; Supplemental Fig. S2). To determine if this pattern of electrolyte leakage following leafhopper infestation was independent of host, we tested lettuce plants and continued to observe higher electrical conductivity estimates at 24, 48, and 72 hpi (*P* < 0.0001; Fig. 1b). Electrical conductivity values were not measured in response to aphids on lettuce.

### *S. enterica* populations naturally accumulate at the tips of tomato leaflets

**Figure 2:**
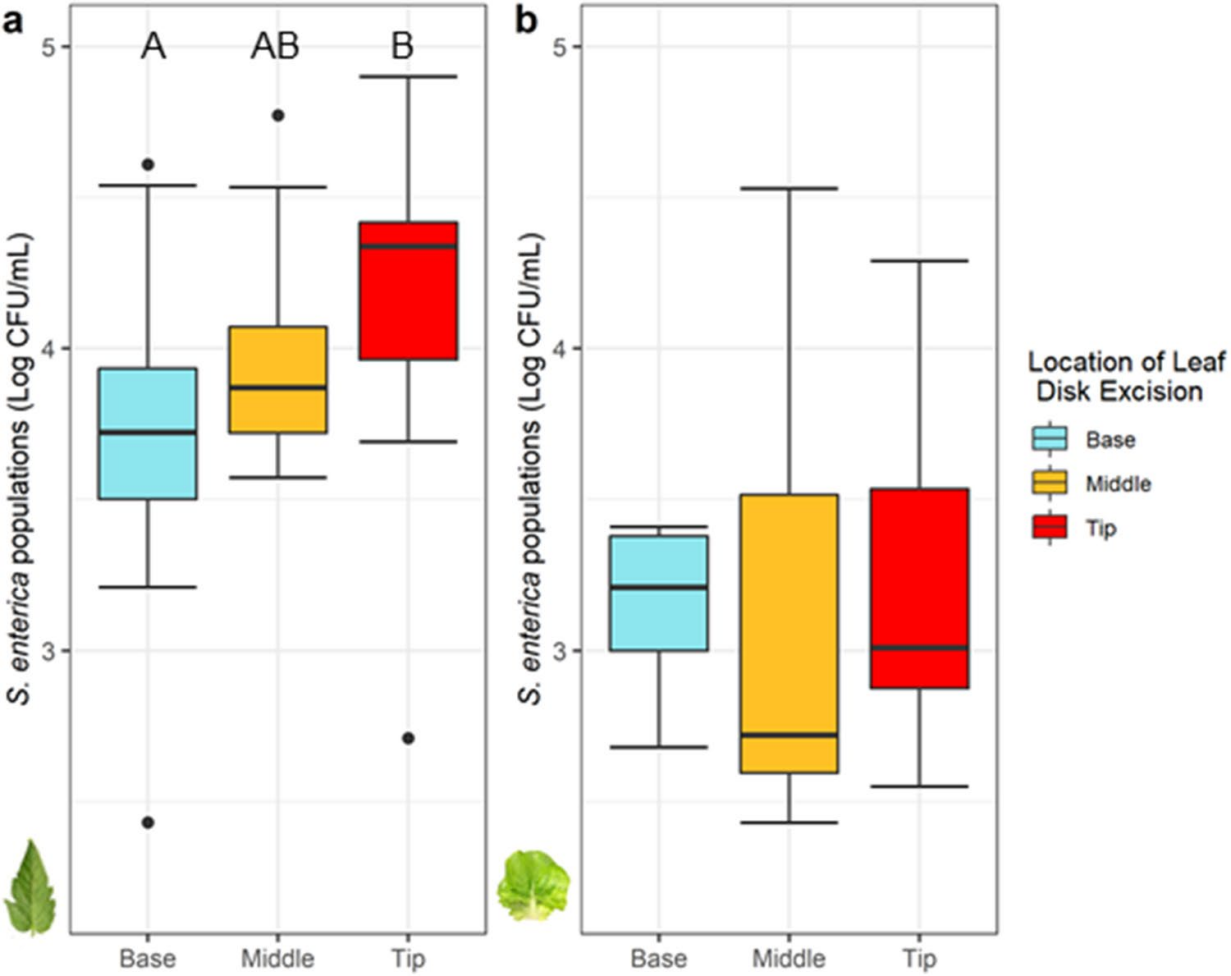
Tomato leaf architecture impacts the distribution of *S. enterica* populations, unlike lettuce. *Salmonella enterica* populations were significantly greater at the tip of tomato leaflets than the base (**a**) (*P* < 0.05), whereas lettuce leaves have a uniform bacterial distribution (**b**). Leaf discs were excised from pre-determined locations from the basal (blue), middle (orange), and tip (red) regions of leaves. Letters above boxplots indicate significant differences between treatment groups within each experiment (*P* < 0.05), as detected by a one-way ANOVA. Singular dots represent an outlier point.

To better understand how leafhopper feeding alters the distribution of *S. enterica* in the phyllosphere, we examined how bacterial populations changed across the leaf surface. First, the natural distribution of *S. enterica* on lettuce and tomato plants was determined (Fig. 2). Tomato leaflets supported significantly higher *S. enterica* populations at the tip of leaflets, compared to samples measured from the basal regions (*P* < 0.0007; Fig. 2a). Measurements of electrical conductivity at the base of tomato leaflets were higher, but not significantly different than samples collected in the middle or tip regions of leaflets (*P* = 0.0626; Supplemental Fig. S3a). To test whether *S. enterica* population distribution across the tomato leaflet is influenced by gravity, a complementary experiment was designed to disrupt the natural tendency of leaflets to droop and consequentially result in liquid collecting on leaflet tips. When leaflets were placed in a more upright position (65° upward angle) after the dip-inoculation, the highest accumulation of bacterial populations shifted to the base (*P* = 0.0068; Supplemental Fig. S4), whereas measured electrical conductance remained higher, but not significantly different, at the basal region of the leaf (*P* = 0.061; Supplemental Fig. S5). On lettuce, *S. enterica* populations remained somewhat uniform across the leaf (*P* = 0.87; Fig. 2b) while the base of lettuce plants exhibited significantly higher electrical conductivity than the middle or tip regions (*P* < 0.001; Supplemental Fig. S3b). With improved knowledge of where *S. enterica* preferentially colonizes the phyllosphere, we could examine if leafhopper infestation alters bacterial distribution.

### *M. quadrilineatus* infested tomato leaflets had the greatest electrical conductivity and enhanced *S. enterica* populations

**Figure 3:**
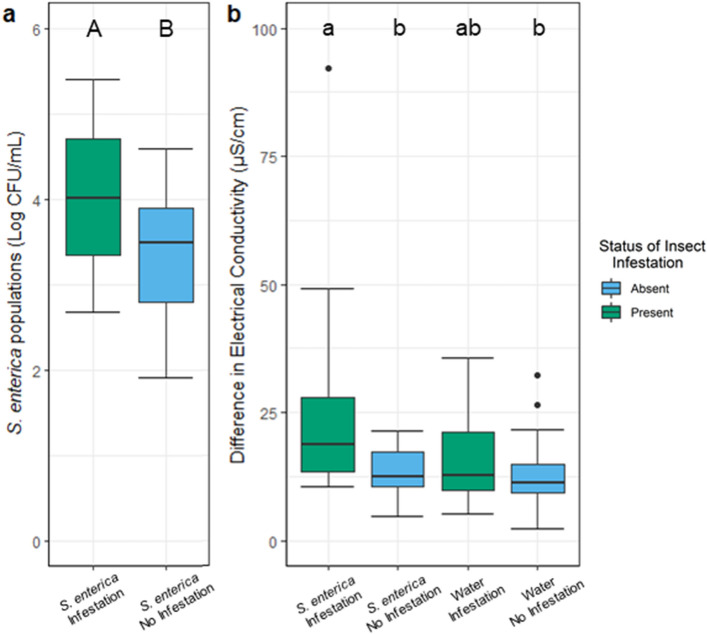
*Macrosteles quadrilineatus* infestation on *S. enterica* inoculated tomato plants led to heightened bacterial populations (**a**) and electrical conductivity (**b**) than plants absent insect infestation. Plants inoculated with either *S. enterica* or water (mock) were infested by adult *M. quadrilineatus* or remained absent of insects. Empty clip cages were applied for treatment groups with no infestation. Electrical conductance was calculated by subtracting the final from the initial measurement for damaged and undamaged leaf discs and were used to evaluate the extent of electrolyte leakage over six hours. Each treatment group contains combined data from the tip, middle, and basal regions of leaves. *Salmonella enterica* populations were measured on water inoculated leaves but yielded 0 CFU and were thus excluded from the figure. Letters above boxplots indicate significant differences between treatment groups within each experiment (*P* < 0.05) as detected by a one-way ANOVA. Singular dots represent an outlier point.

**Figure 4:**
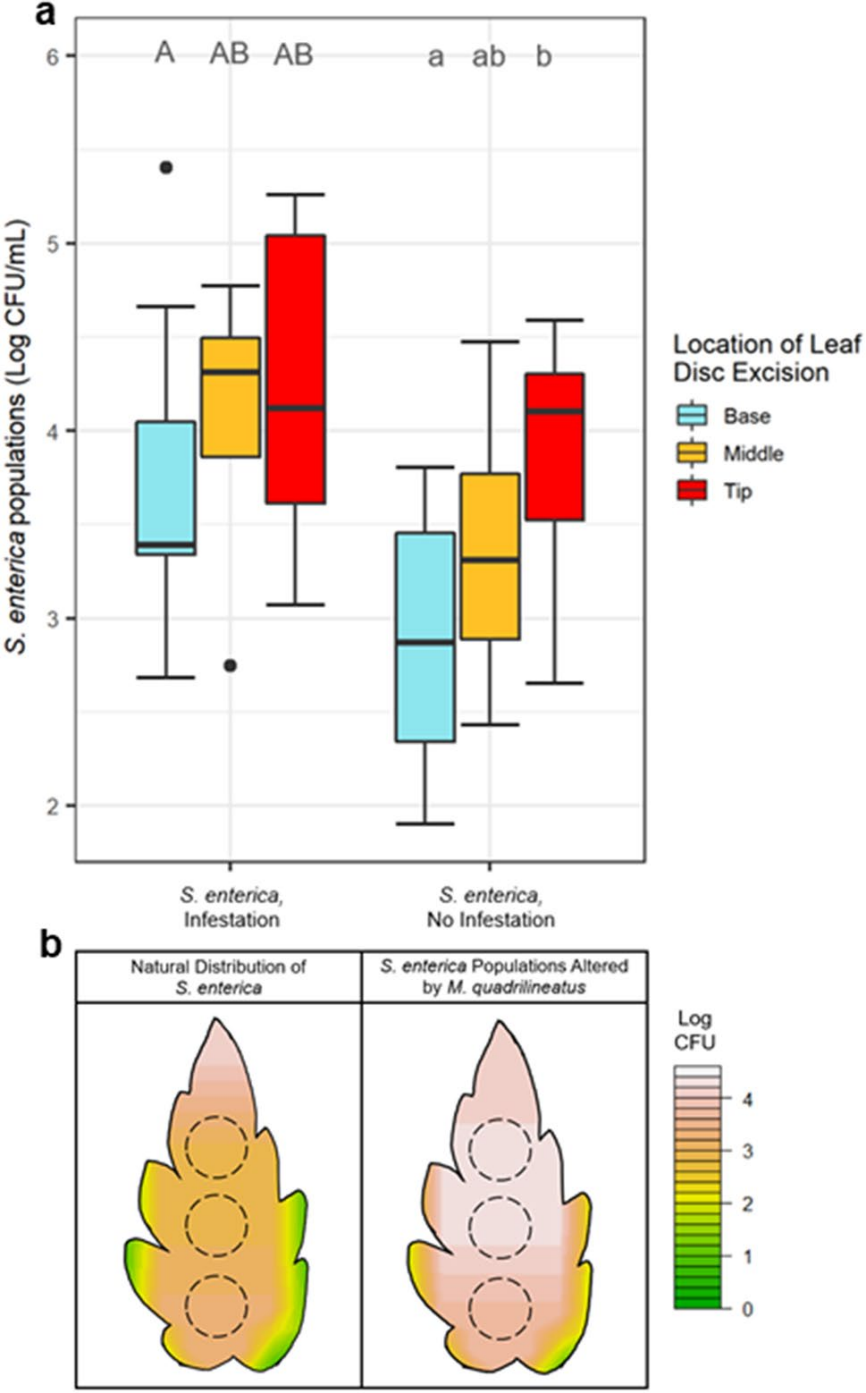
Adult *M. quadrilineatus* infestation on *S. enterica* inoculated tomato plants resulted in a redistribution of bacterial populations. In the absence of insects, *S. enterica* populations are significantly higher at the tips of leaflets (*P* < 0.05); however, after insect infestation, *S. enterica* populations are approximately uniform across the tip and middle (**a**, **b**) regions. The 2 X 2 factorial experiment included *S. enterica* or water inoculated plants that were either infested, or not infested by *M. quadrilineatus*. Empty clip cages were applied for treatment groups with no infestation. Leaf discs were excised from the tip, middle and basal regions of leaflets. *Salmonella enterica* populations were also measured on water inoculated leaves but yielded 0 CFU, and were thus excluded from the figure. An interpolation graph was created to depict the shift in bacterial populations (Log CFU) over a 72 h post-infestation period. Singular dots represent an outlier point. Letters above boxplots indicate significant differences between leaf treatment groups within an insect infestation treatment (*P* < 0.05), as detected by a one-way ANOVA.

To test the hypothesis that cellular damage, prompted by leafhopper intracellular penetration, facilitates fundamental changes in the phyllosphere, we measured *S. enterica* populations in response to *M. quadrilineatus* infestation (Fig. 3). After 72 hpi, *S. enterica* populations were approximately half a log higher on tomato plants infested with leafhoppers than *S. enterica* inoculated plants without insects, consistently indicating that leafhopper infestation enhances *S. enterica* populations (*P* < 0.0001; Fig. 3a). Similarly, plants infested by leafhoppers and contaminated with *S. enterica* exhibited significantly greater estimates of electrolyte leakage than plants without insects (*P* = 0.0020; Fig. 3b), and had higher, but not significantly different, electrical conductivity measurements than infested plants treated with water only. Infested plants had a roughly uniform distribution across the middle and tip regions of leaflets indicating a shift in expected natural bacterial populations. Although the *S. enterica* population was similar at the tip for either infested or leaves without insects, the base and middle locations of infested leaflets had significantly higher *S. enterica* than the same locations on uninfested leaflets (*P* < 0.005; Fig. 4a, b), suggesting that insect activity increased the local *S. enterica* populations in these regions of the leaflet. Electrical conductivity was not significantly different across a leaflet within any treatment group and was similar between the tip, middle, and basal regions of infested *S. enterica* tomato plants (*P* > 0.05; Supplemental Fig. S6). Greater cellular damage on infested, *S. enterica* inoculated leaflets prompted an additional set of experiments to determine whether *M. quadrilineatus* feeding and resting preference was influenced by *S. enterica* or water-inoculated leaves. Measurements of *S. enterica* populations and electrical conductivity within a randomized block design were similarly measured for *M. persicae,* yet no significant differences between infested and uninfested plants were observed (*P* > 0.05; Supplemental Fig. S7).

### Adult *M. quadrilineatus* prefer water inoculated surfaces, over those inoculated with *S. enterica*

**Figure 5:**
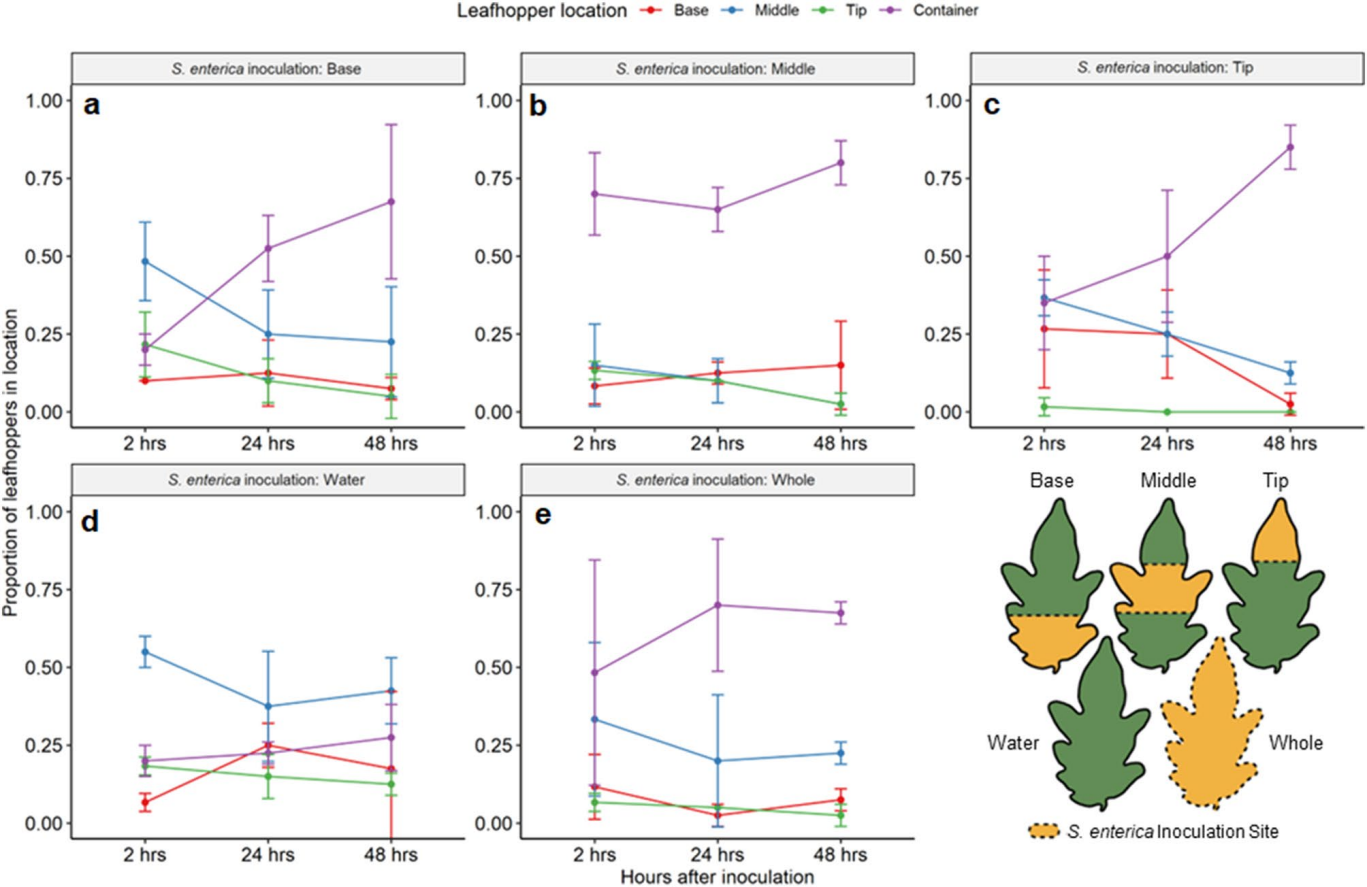
The proportion of *M. quadrilineatus* that emigrate away from leaflets inoculated with *S. enterica* increase over 48 h. Tomato leaflets were inoculated with *S. enterica* exclusively at the basal (**a**), middle (**b**) or tip (**c**) regions or were entirely submerged during inoculation (Whole; **e**). Similarly, one group of tomato leaflets were entirely inoculated by water (Water; **d**). One hour after *S. enterica* inoculation, five leafhoppers were placed in a container encasing one tomato leaflet, still attached to the plant. Observations were taken 2, 24, and 48 h after the initial infestation period. The proportion of insects within each treatment represent means from across three experimental replicates between four plants (N = 60 adult *M. quadrilineatus*).

When provided a choice, adult *M. quadrilineatus* discriminated between non-plant and plant surfaces over a 2-h period, landing more frequently on water inoculated areas than on *S. enterica* inoculated areas (*P* < 0.005; Supplemental Fig. S8). When exposed to partially or entirely inoculated leaflets for a greater duration, *M. quadrilineatus* were observed to explore leaf surfaces at 2-h post infestation but migrated away from leaflets and onto the experimental cage after 48-h of exposure to *S. enterica* (*P* < 0.001; Fig. 5). Similar to *M. quadrilineatus*, sets of *M. persicae* were also exposed to inoculated tomato leaflets over a 72 h experimental interval. While a pattern of emigration from inoculated leaflets and towards the cage of the experimental arena emerged over the 72-h period of infestation, apterous *M. persicae* indicated no significant substrate preference (*P* > 0.05; Supplemental Fig. S9).

### Salivary sheath distribution is dependent upon *S. enterica* presence

**Figure 6:**
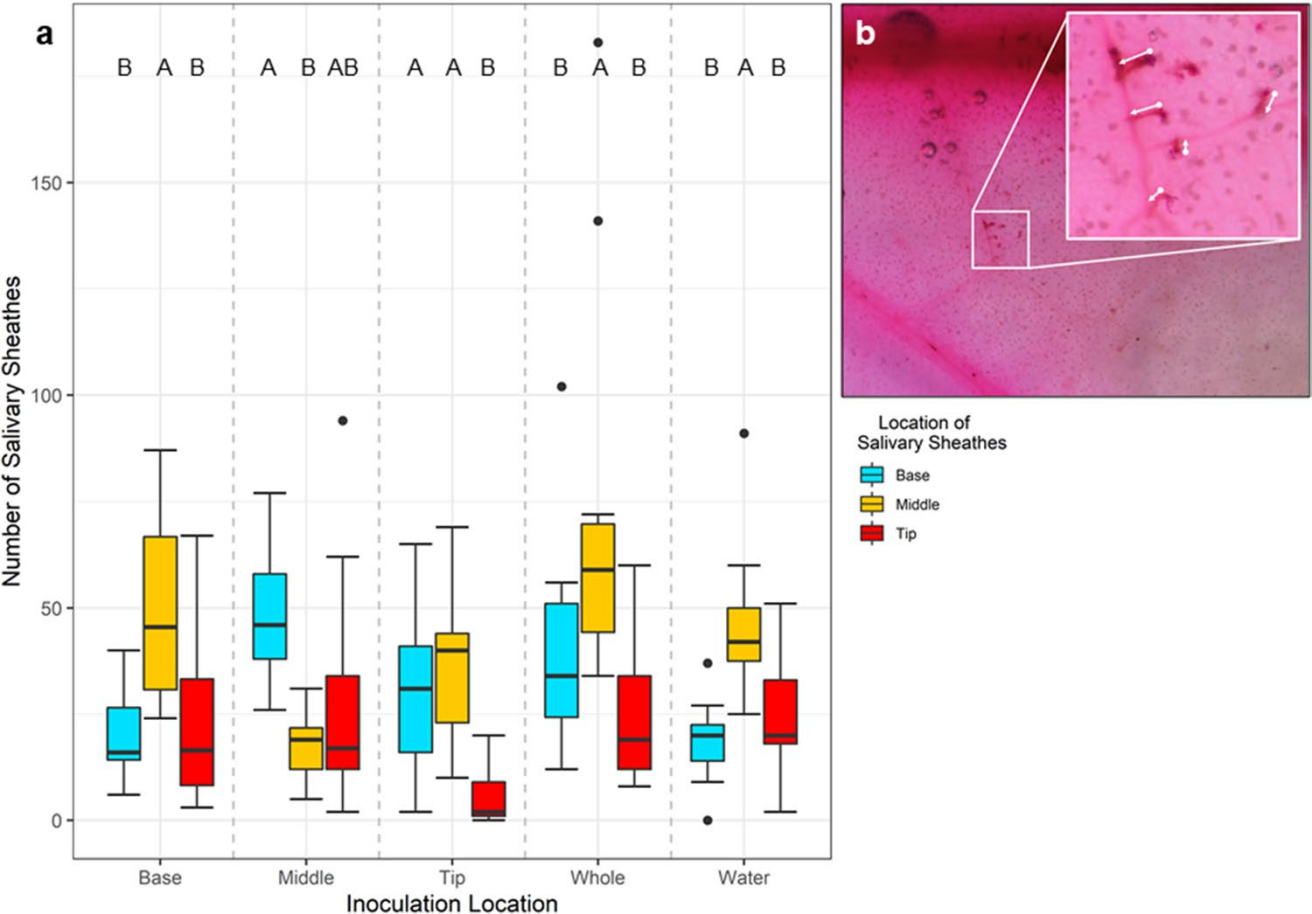
Salivary sheath distribution across tomato leaflets in response to *S. enterica* inoculations applied to different regions of plants, excluding those found on the primary and secondary veins. Tomato leaflets were inoculated with *S. enterica* exclusively at the basal, middle or tip regions, or were entirely submerged during inoculation (Whole). Similarly, one group of tomato leaflets were entirely inoculated by water (Water). One hour after *S. enterica* inoculation, five adult *M. quadrilineatus* were placed in a container encasing one tomato leaflet still attached to the plant. Leaflets were extracted 72 h post *S. enterica* inoculation and were subjected to staining and clearing procedures to count total salivary sheaths (**a**). Post clearing and staining procedures, salivary sheathes appeared as dark red in contrast to the pink leaflets (**b**). Salivary sheathes from three experimental replicates (n = 60 leaflets) are combined and represented above. Letters above boxplots indicate significant differences between leaf treatment groups within an insect infestation treatment (*P* < 0.05).

To further define *M. quadrilineatus’* preferred feeding sites in relation to the presence of *S. enterica*, the presence of salivary sheathes across water and *S. enterica* inoculated leaflets were observed. Across all inoculation treatment groups, salivary sheathes were observed to be significantly less abundant on primary and secondary veins indicating a predominant preference for tertiary, or lesser, veins (*P* < 0.0001; Supplemental Fig. S10). As a result, salivary sheathes located on primary and secondary veins were excluded from statistical comparisons of salivary sheath distribution across the basal, middle and tip regions of uniquely inoculated leaflets. Salivary sheathes were most regularly found on the middle of leaflets inoculated at the base or tip, and on leaflets inoculated entirely with *S. enterica* or water (*P* < 0.0001; Fig. 6). However, inoculation of *S. enterica* exclusively on the middle portions of leaflets, their preferred feeding location, resulted in a shift of salivary sheath distribution as significantly more salivary sheathes were found at the base of leaflets than the middle (*P* = 0.0237; Fig. 6). This finding demonstrates that even limited presence of *S. enterica* on a leaflet alters the preferred probing/feeding locations of adult *M. quadrilineatus*.

## Discussion

The now frequent, reoccurrence of foodborne illness cases associated with consumption of fresh produce requires an in-depth assessment of environmental factors that increase the risks of continued outbreaks. In our current study, we examined the population dynamics of a foodborne pathogen through an entomological perspective, analyzing the tri-trophic interactions between *S. enterica*, plants, and phytophagous insects. Specifically, we investigated how changes in the phyllosphere resulting from unique insect feeding styles, impacted the longevity and persistence of *S. enterica* populations on the leaf surface.

Previous literature demonstrated that insects can manipulate human enteric bacterial pathogen populations directly, and indirectly. Within poultry dominated environments, cockroaches may mechanically transmit *S. enterica* by traversing from contaminated egg surfaces to uncompromised substrates, consequently facilitating the movement of bacteria^25^. Seaweed flies, intimately associated with decaying and pathogenic seaweed beds, excrete viable bacterial populations within intertidal zones, enhancing the potential transmission of *E. coli*^26^. Despite *S. enterica* populations decreasing by 2 logs over a 13-day period upon tomato hosts, *M. quadrilineatus* enhances transmission of *S. enterica* from contaminated leaves to clean leaves or adjacent plants within an agriculturally-relevant context^8,13^. Furthermore, excretion of viable *S. enterica* from *M. quadrilineatus* has also been documented^27^. Yet, how phytophagous insects influence this increase of *S. enterica* persistence on leaves remains mostly unexamined.

In earlier studies, we observed that only *M. quadrilineatus* infestation led to an increase in *S. enterica* persistence, but no observed benefit occurred following *M. persicae* infestation^8^. The findings of this investigation point towards differences between the inter- and intracellular penetrative styles of feeding between these two taxa, and the resulting effects these styles may hold for *S. enterica* population dynamics within the phyllosphere. While both insects possess similar mouthpart structures, collectively referred to as stylets, their modes of reaching vascular tissues are very distinct. Aphids, or intercellular feeders, begin probing at the junction of two epidermal cells and guide their stylet through intercellular spaces in the mesophyll and towards vascular bundles^28^. Leafhoppers, considered as intracellular feeders, similarly begin feeding at a cell junction, but distinctly pierce through leaf mesophyll to reach the phloem^29^. Comparisons of the electrical conductivity response of leaflets infested by inter- and intracellular penetration revealed that *M. quadrilineatus* infestation elicits a greater magnitude of electrolyte leakage, and consequently greater cellular damage than *M. persicae* on tomato plants (Fig. 1; Supplemental Fig. S1). Furthermore, our current study demonstrated that plants contaminated by *S. enterica* and infested with *M*. quadrilineatus had the highest overall populations of bacteria and resulted in the greatest magnitude of electrolyte leakage (measured as electrical conductance) (Fig. 3a-b). In addition to their distinct feeding behaviors, leafhoppers possess a stylet bundle 5-times wider than those found on aphids^30,31^. To compensate for the lesser stylet, we investigated the influence of higher aphid populations in a complementary experiment, yet found no measurable impact on enhanced electrolyte leakage, or cellular damage (Supplemental Fig. S2). Taken together, the wider stylet paired with intracellular lacerating types of feeding behavior by *M. quadrilineatus* may partially explain the enhanced magnitude of cellular damage on the phyllosphere of tomato plants (Fig. 1). These findings lead us to conclude that cellular damage induced by *M. persicae* probing behaviors does not manipulate the phyllosphere to the same extent as *M. quadrilineatus*.

As previously mentioned, *S. enterica* and *M. quadrilineatus* co-habitation on the same leaflet resulted in higher *S. enterica* populations and measured electrolyte leakage (aka cellular damage) compared to water inoculated leaflets with or without insects (Fig. 3a-b). These elevated levels of cellular damage are likely the result of greater probing frequencies and may indicate an unfavorable feeding environment for the insect, prompting them to more frequently probe and search for alternative food sources. Previous studies identified clusters of gustatory neurons, which when combined, functionally create taste receptors within insects^32^. When encountering food contaminated by lipopolysaccharides (LPS), a ubiquitous component found on gram-negative bacterial cells, *Drosophila melanogaster* not only avoids *E. coli-*contaminated foods but also commence a hygienic grooming regimen^33^. This prompted behavior suggests that some insects can discriminate between LPS contaminated and non-contaminated food sources via gustatory cues. Although many of these studies focus on insects with sponging-sucking mouthparts, such as flies, a genome analysis identified both odorant and gustatory receptor genes in aphid and mosquito genomes, both of which possess piercing-sucking mouthparts comparable to that of *M. quadrilineatus*^34^. In our experiments where we confined *M. quadrilineatus* and *S. enterica* together in more proximal environments, we propose that the adult leafhoppers could encounter higher traces of LPS and may modify their normal feeding behavior as a consequence. Due to the restricted movement in these instances, we surmise the heightened magnitude of electrolyte leakage is driven by a constant search for a non-contaminated substrate and thus, heightened occurrences of probing for a new food source on *S. enterica* inoculated plant (Fig. 3b). To further evaluate whether *S. enterica* presence alters *M. quadrilineatus*’ movement, we provided *M. quadrilineatus* with contaminated (*S. enterica*) and non-contaminated (sterile water) tomato leaflet surfaces and monitored their resting or feeding locations every 15 min thereafter for over a two-hour period. Throughout the time course of these observational experiments, a pattern of substrate discrimination occurred (Supplemental Fig. S8). Most insects initially landed on the plastic container housing the experiment, but over time began to immigrate more often to water-inoculated surfaces than those with *S. enterica*. In a complementary experiment, adult *M. quadrilineatus* exposed to tomato leaflets inoculated at either tip or basal regions of leaves similarly preferred water inoculated regions at 2 h post exposure, but predominantly emigrated to the experimental container walls after 48 h (Fig. 5). Altogether, leaflets entirely or partially inoculated with *S. enterica* were less frequently visited at the last time point (48 h post infestation), whereas leaflets inoculated solely with water were occupied throughout the experiment. Contrasting this behavior, apterous *M. persicae* exhibited no preference between *S. enterica* or alternative surfaces (Supplemental Fig. S9). This lack of substrate preference may result from the largely sessile lifestyle of aphids, in contrast to more mobile and alate leafhoppers. These avoidance behaviors by *M. quadrilineatus* in response to *S. enterica* inoculated leaflets suggest a capability of recognizing contaminated substrates similar to the responses described for *D. melanogaster*.

To evaluate the extent by which *M. quadrilineatus* might influence the distribution of bacterial populations across leaflets, we first defined the distribution of *S. enterica* and the magnitude of electrolyte leakage across tomato and lettuce leaves in the absence of any insects. Morphological features between pre-reproductive lettuce and tomato plants are vastly distinct and were hypothesized to impact the distribution of bacterial populations and electrolyte leakage. In our study, the leaf tips were the lowest positioned part of tomato leaflets and exhibited half a log higher *S. enterica* populations in comparison to basal regions (Fig. 2a). Here again, the nominal architecture of tomato leaves results in a natural ‘drooping’ of fully expanded leaf tips. In a complementary experiment, tomato leaflets were modified to reverse this normal positioning of leaf tips to basal regions, and we did observe a corresponding re-distribution of *S. enterica* where accumulations were enhanced on basal portions of leaves (Supplemental Fig S4). These findings suggest that during the application of an aqueous solution—such as contaminated irrigation water or even foliar-applied crop inputs—factors including gravitational force may influence aggregations of aqueous solutions on leaves^35^. This suite of findings identified leaf positioning and morphology, in conjunction with gravitational forces, as dominant influences of *S. enterica* population distribution across tomato leaflets while unaffecting the degree of electrical conductivity estimates, or associated electrolyte leakage of leaf electrolytes (Supplemental Fig. S3b). Despite *S. enterica* populations being highest at the tips of unaffected leaflets, bacterial populations were comparable at the tip and middle portions of leaflets only after adult *M. quadrilineatus* infestation, suggesting an insect mediated influence (Fig. 4). To this finding, we hypothesized that leafhopper feeding is not uniform or homogeneous across whole leaflets and that the distribution of leaf vascular bundles may influence where adult leafhoppers find preferential feeding sites. The diameter of primary and secondary angiosperm vascular bundles typically narrows from the base to the tip of leaves, presumably to maximize the efficiency of hydraulic conductivity using adhesive and cohesive forces^36,37^. This natural tapering of vascular structures at the tips of leaves provides piercing-sucking insects with some limitations in the number of ideal feeding locations and we hypothesize that the variation in the dendritic nature of leaf venation may alter the distribution of *M. quadrilineatus* feeding sites, explaining the higher *S. enterica* populations in the middle of infested tomato leaflets^38^. In addition to frequently observing leafhoppers in middle portions of leaflet regions, salivary sheathes were also predominantly found in similar regions of water-inoculated leaflets indicating preferences for these vascular bundles across leaflets (Fig. 6). Despite being their preferred feeding site, *S. enterica* inoculation at the middle of leaflets appeared to influence adult *M. quadrilineatus* towards feeding at the non-contaminated basal and tip regions, away from the *S. enterica* middle regions (Fig. 5). Similarly, leaflets partially inoculated at the base and tip had the least amount of salivary sheathes at their base and tip, respectively. This consistent pattern of probing avoidance of contaminated regions suggests that *M. quadrilineatus* may exhibit discriminatory behaviors against leaflets where *S. enterica* was present, indicating that even limited exposure to *S. enterica* holds potential to alter natural feeding behaviors as seen on water inoculated leaflets.

**Figure 7:**
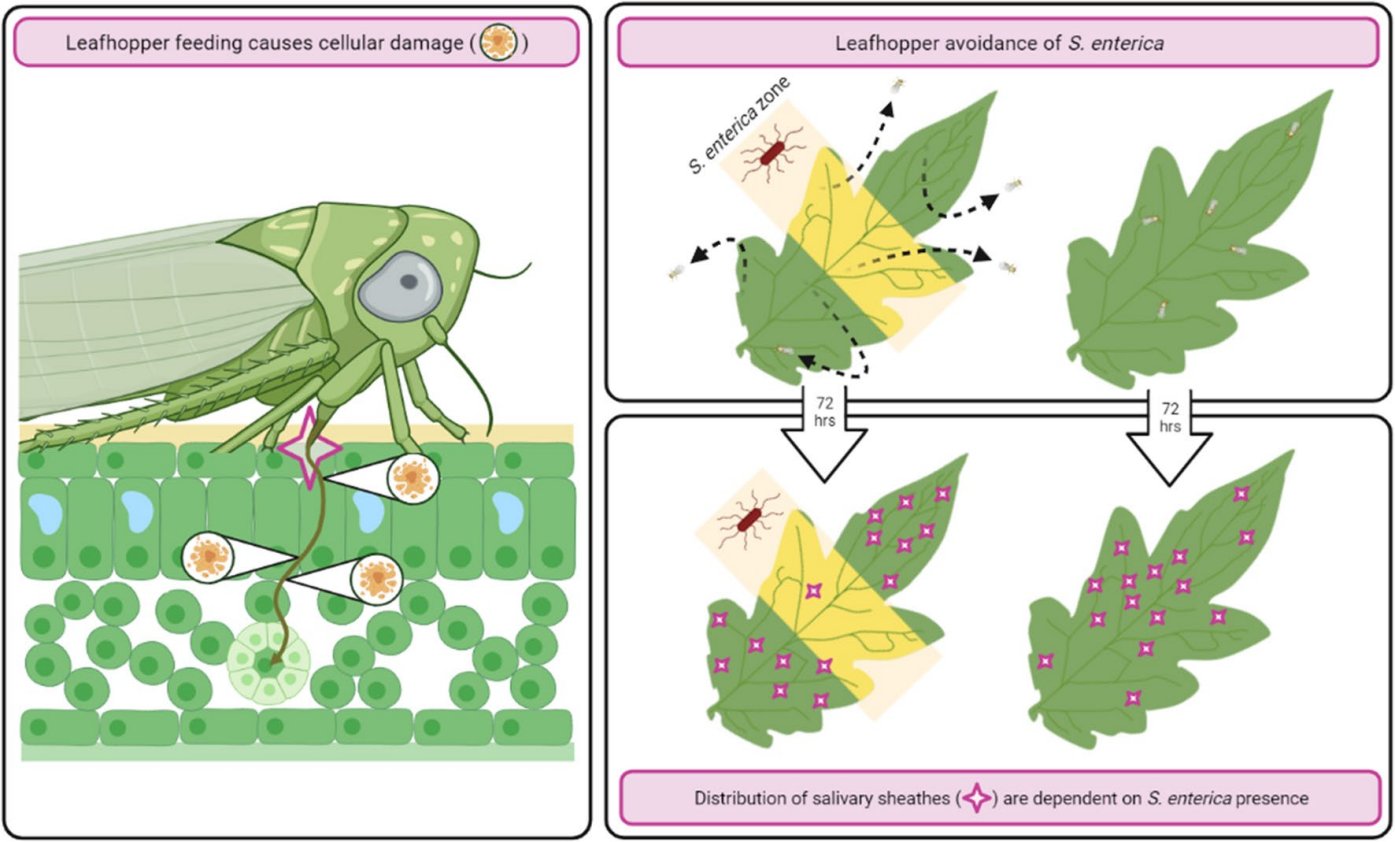
The presence of *S. enterica* alters *M. quadrilineatus* feeding behaviors. Infestation by *M. quadrilineatus* on *S. enterica* inoculated tomato plants resulted in significantly greater rates of localized cellular damage and bacterial populations than uninfested leaflets (left panel). Over a 48-h period of infestation, cohorts of *M. quadrilineatus* migrated away from tomato leaflets with partial, or entire, *S. enterica* inoculation (top). Cleaning and staining procedures 72-h afterwards demonstrated that zones of bacterial inoculation contained the least amount of salivary sheathes indicating an aversion to *S. enterica* within the phyllosphere (bottom). Image created through Biorender (biorender.com).

Although *M. quadrilineatus* exhibited avoidance behaviors of partially inoculated leaflets, their mobile lifestyle illustrates their potential as a biological multiplier for *S. enterica*. During their exposure to partially inoculated leaflets, salivary sheathes were identified at the base, middle and tip, although nonuniformly, suggesting an exploratory behavior (Fig. 6). This movement across *S. enterica* contaminated leaflets and the subsequent aversion suggest a likelihood for emigrating to alternative food sources (Fig. 7). Logically, if *M. quadrilineatus* have previously encountered *S. enterica* contaminated leaves or plants, then mechanical transmission of bacteria could further exacerbate the likelihood of *S. enterica* dissemination within contaminated agricultural crops and promote the possibility of food borne outbreaks.

Within this study, we aimed to characterize insect feeding behaviors which could directly enhance *S. enterica* populations on tomato leaflets. Although we directly focused on cellular damage by stylet penetration, a suite of other phenomena (i.e. honeydew production and plant immunity regulation) occurring in tandem necessitate further investigation. While these biological factors likely co-occurred, we identified prominent insect-mediated interactions involving cellular damage, unique insect feeding behaviors, and *S. enterica* populations, thereby demonstrating intracellular stylet penetration by *M. quadrilineatus* as a beneficial insect behavior for *S. enterica* persistence. Furthermore, we demonstrated that plant morphology directs the distribution of bacterial populations when dispersed aqueously yet may be manipulated in the presence of *M. quadrilineatus* due to increased stylet probing at preferred feeding sites. Although our results were collected under laboratory conditions, our findings elucidate how insects interact within the phyllosphere, and in turn, influence *S. enterica* population dynamics.

## Supplementary Information

Supplementary Information.
