# Supplementary material for: Salmonella enterica changes Macrosteles quadrilineatus feeding behaviors resulting in altered S. enterica distribution on leaves and increased populations
Source: Sci Rep. 2022 May 20;12:8544. doi: 10.1038/s41598-022-11750-3 (PMC9122940; doi:10.1038/s41598-022-11750-3)
Supplement: Supplementary file 1 — Supplementary Information. [file 41598_2022_11750_MOESM1_ESM.pdf]

Supplementary Information

Salmonella enterica changes *Macrosteles quadrilineatus* feeding behaviors resulting in altered *S. enterica* distribution on leaves and increased populations.

Authors

Victoria L. Harrod (lason@wisc.edu)[A], Russell L. Groves (rgroves@wisc.edu)[A], Ellie G. Guillemette (eguillemette@wisc.edu)[B], Jeri D. Barak (jeri.barak@wisc.edu)[B]

***Corresponding Author**

*Jeri D. Barak (jeri.barak@wisc.edu)[B]

Affiliations

[A] Department of Entomology, University of Wisconsin - Madison, Madison, Wisconsin, USA
[B] Department of Plant Pathology, University of Wisconsin - Madison, Madison, Wisconsin, USA

pathogen, biological multiplier)

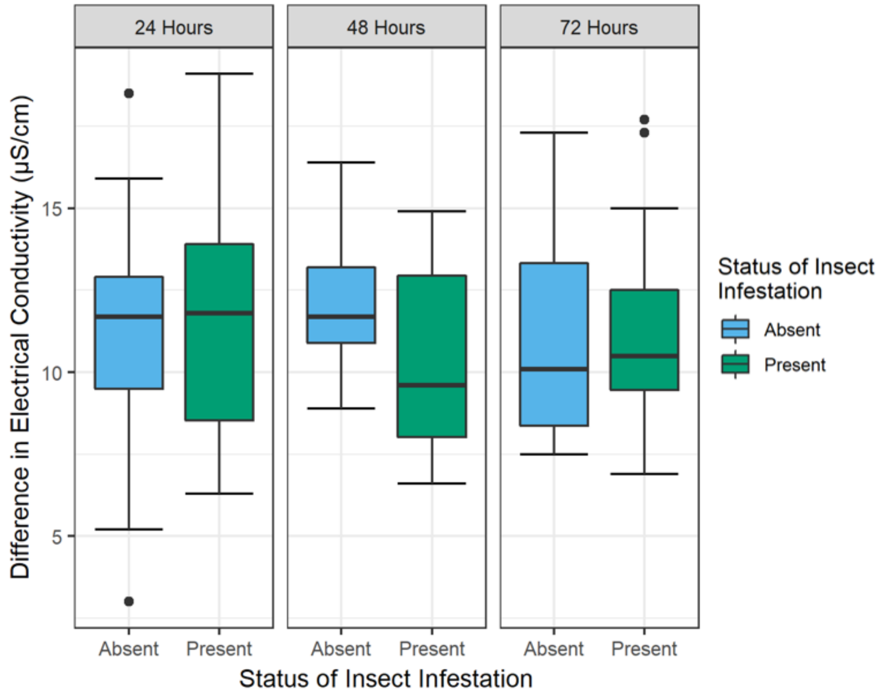

Supplemental Figure S1. **Influence of *Myzus persicae* infestation upon electrical conductance measured at 24-, 48- or 72-hours post-infestation**. Three clip cages were fastened onto a middle leaf, each containing one aphid (green), and three additional clip cages remained empty (blue) on the opposite tomato leaf. Measures of electrical conductance were calculated by subtracting the final from the initial measurement for damaged and undamaged leaf discs and were used to evaluate the extent of electrolyte leakage over six hours. A student's t-test was used to assess significance between samples from infested, or non-infested clip cages. Singular dots represent an outlier point.

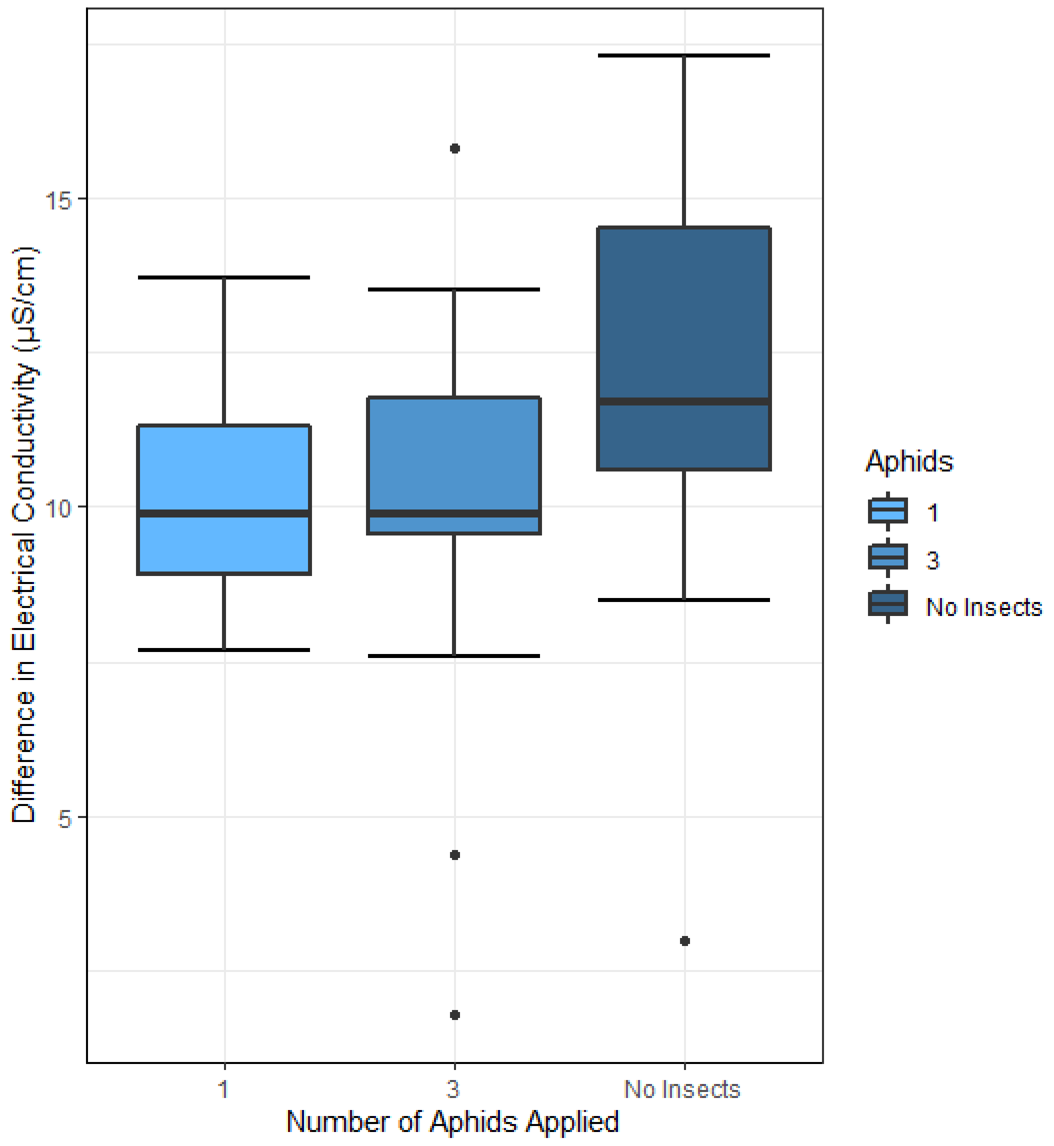

Supplemental Figure S2. Regardless of initial infestation density, populations of *Myzus persicae* allowed to feed for 72 hours did not elicit an increase in electrical conductance. Clip cages were fastened onto the middle of a leaflet containing one (light blue) or three (blue) aphids or remained empty (dark blue). Measures of electrical conductance were calculated by subtracting the final from the initial measurement for damaged and undamaged leaf discs and were used to evaluate the extent of electrolyte leakage over six hours. A one-way ANOVA was used to assess significance between samples from infested, or non-infested clip cages. Singular dots represent an outlier point.

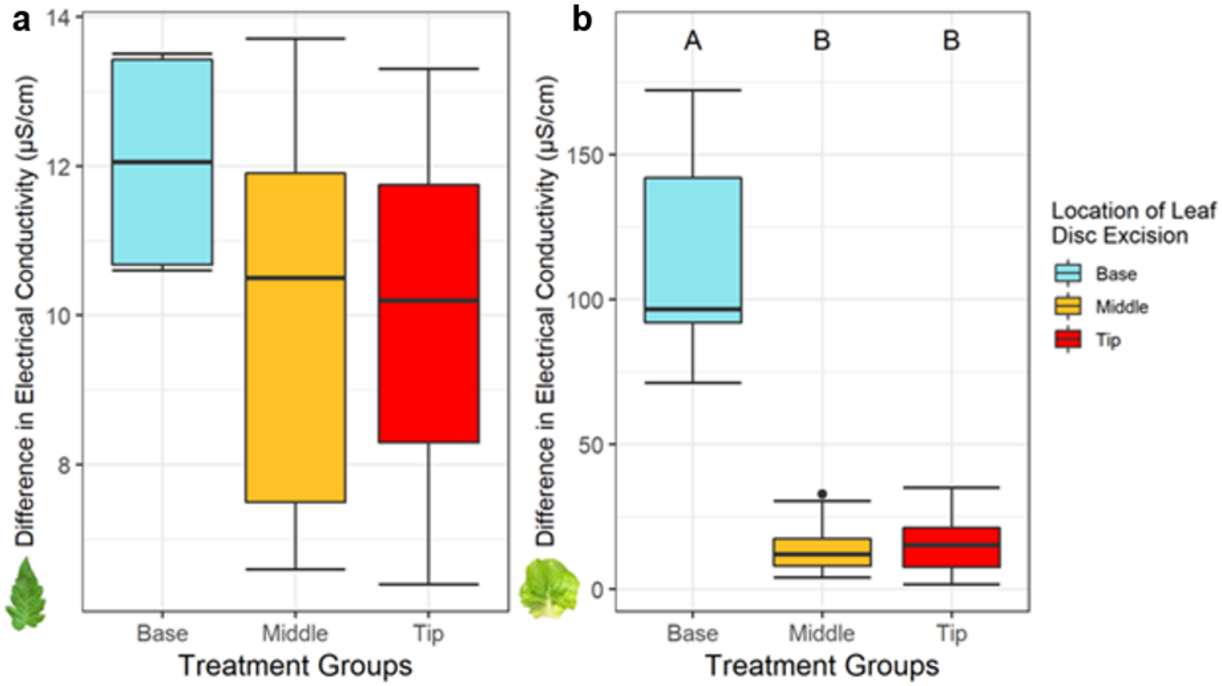

Supplemental Figure S3. Estimates of electrical conductance varied among regions of leaves in both lettuce and tomato. Tomato leaflets had a higher, albeit non-significant estinate of mean electircal conductance at its basal region (Supplemental Fig. S3a), whereas lettuce leaves had a significantly greater electrical conductance at its base (Supplemental Fig. S3b). Leaf discs were excised from pre-determined locations from the base (blue), middle (orange), and tips (red) of leaves (Fig. 3a). Measures of electrical conductance were calculated by subtracting the final from the initial measurement for damaged and undamaged leaf discs and were used to evaluate the extent of electrolyte leakage over six hours. Letters above boxplots indicate significant differences between treatment groups within each experiment ($P < 0.05$), as detected by a one-way ANOVA. Singular dots represent an outlier point.

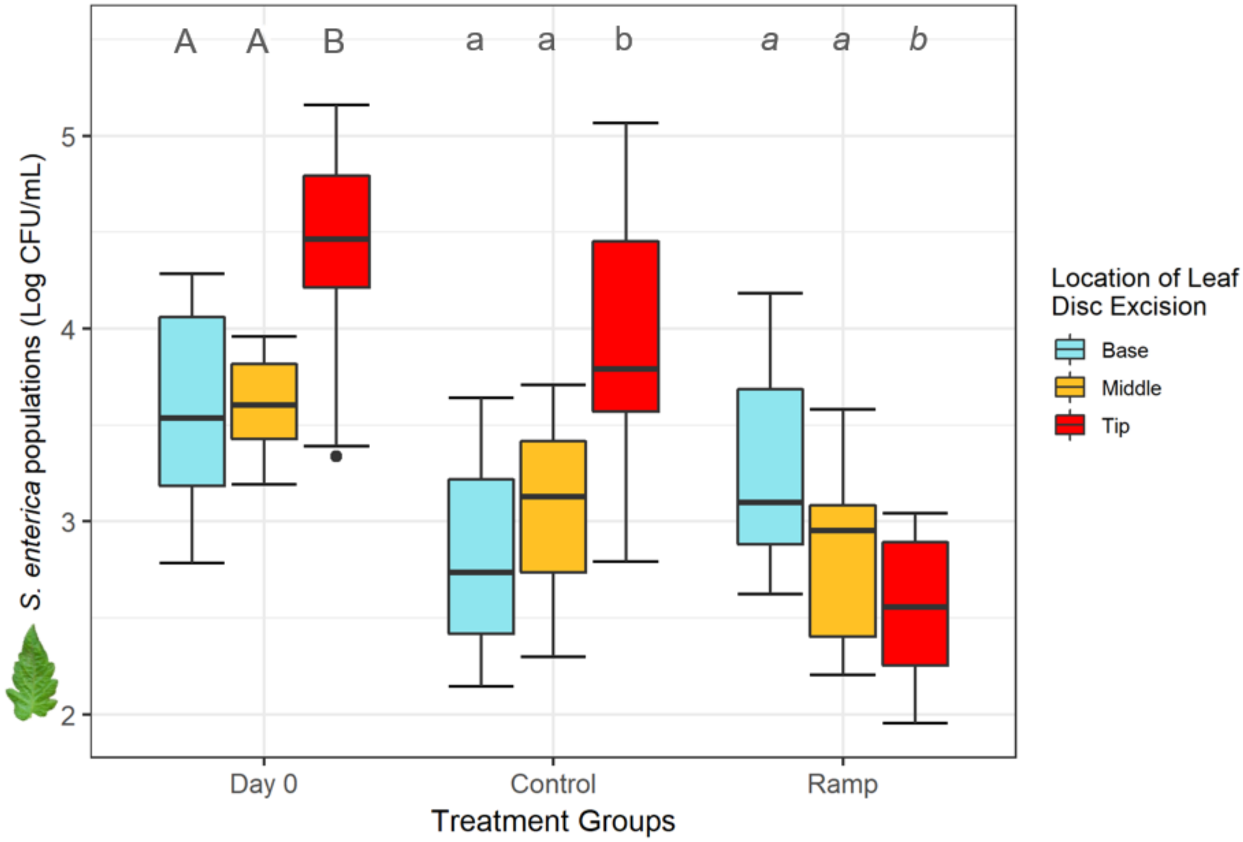

Supplemental Figure S4. The position of tomato leaflets impacts the resulting distribution of *S. enterica* in the phyllosphere. The tips (red) of unmanipulated tomato leaflets (Control & Day 0) support significantly higher *S. enterica* populations than the basal (blue) regions, whereas tomato leaflets facing upward (65º upward angle) had the greatest bacterial populations at its base (blue). Tomato plants were dip inoculated in an *S. enterica* or water solution and immediately placed into position. Control plants were directly placed into empty bins, whereas ramp treated plants were placed into bins with Plexiglass ramps at an approximately 65° upward angle for leaflets to vertically rest upon. After 72 hours, each plant was removed from its bin and measured for *S. enterica* populations across the base, middle or tip regions from among randomly chosen leaflets. *S. enterica* populations were measured on water inoculated leaves but yielded 0 CFU and were thus excluded from the figure. Each treatment group represents 16 biological replicates. Letters above boxplots indicate significant differences between treatment groups within each experiment ($P < 0.05$), as detected by a one-way ANOVA. Singular dots represent an outlier point.

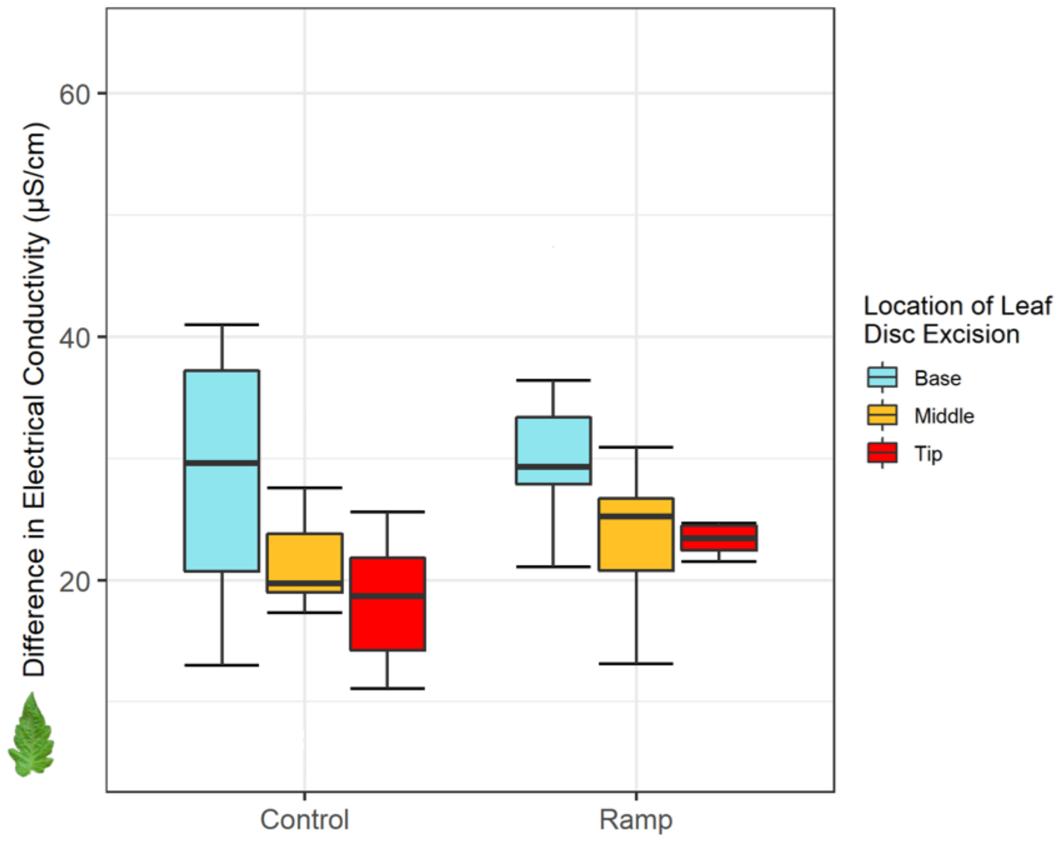

Supplemental Figure S5. Estimates of electrical conductivity did not change across a tomato leaflet in response to altered leaf positions. The base (blue) of tomato leaflets had a higher, but not significantly different extent of measured electrical conductance when compared to estimates at leaf tips (red; $P > 0.05$). Tomato plants were dip inoculated in an *S. enterica* or water solution and immediately placed into position. Control plants were directly placed into empty bins, whereas ramp treated plants were placed into bins with Plexiglass ramps an approximately 65° upward angle for leaflets to vertically rest upon. After 72 hours, each plant was removed from its bin and assessed for electrolyte leakage across the base, middle or tip regions from among randomly chosen leaflets. Data from *S. enterica* and water inoculated plants are both combined and represented in the figure above. Measures of electrical conductance were calculated by subtracting the final from the initial measurement for damaged and undamaged leaf discs and were used to evaluate the extent of electrolyte leakage over six hours. Each treatment group represents 16 biological replicates.

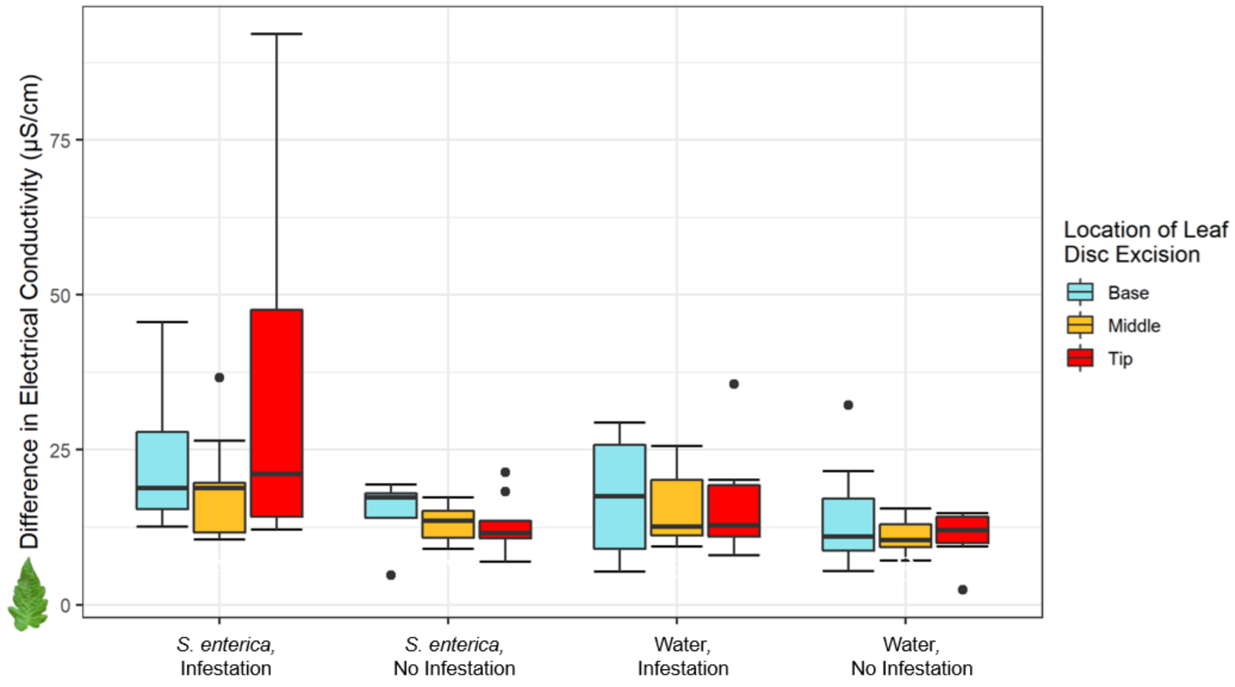

Supplemental Figure S6. Adult *M. quadrilineatus* infestation did not shift the natural magnitude of electrical conductivity on tomato plants. Among treatment groups, estimates of electrolyte leakage (measured as changes in electrical conductivity) was uniform across the basal (blue), middle (orange) and tip (red) regions of tomato leaflets. Salmonella *enterica* or water inoculated plants were either infested or remained un-infested using adult *M. quadrilineatus*. Leaf discs were excised from the tip, middle and basal regions of leaflets. Singular dots represent an outlier point.

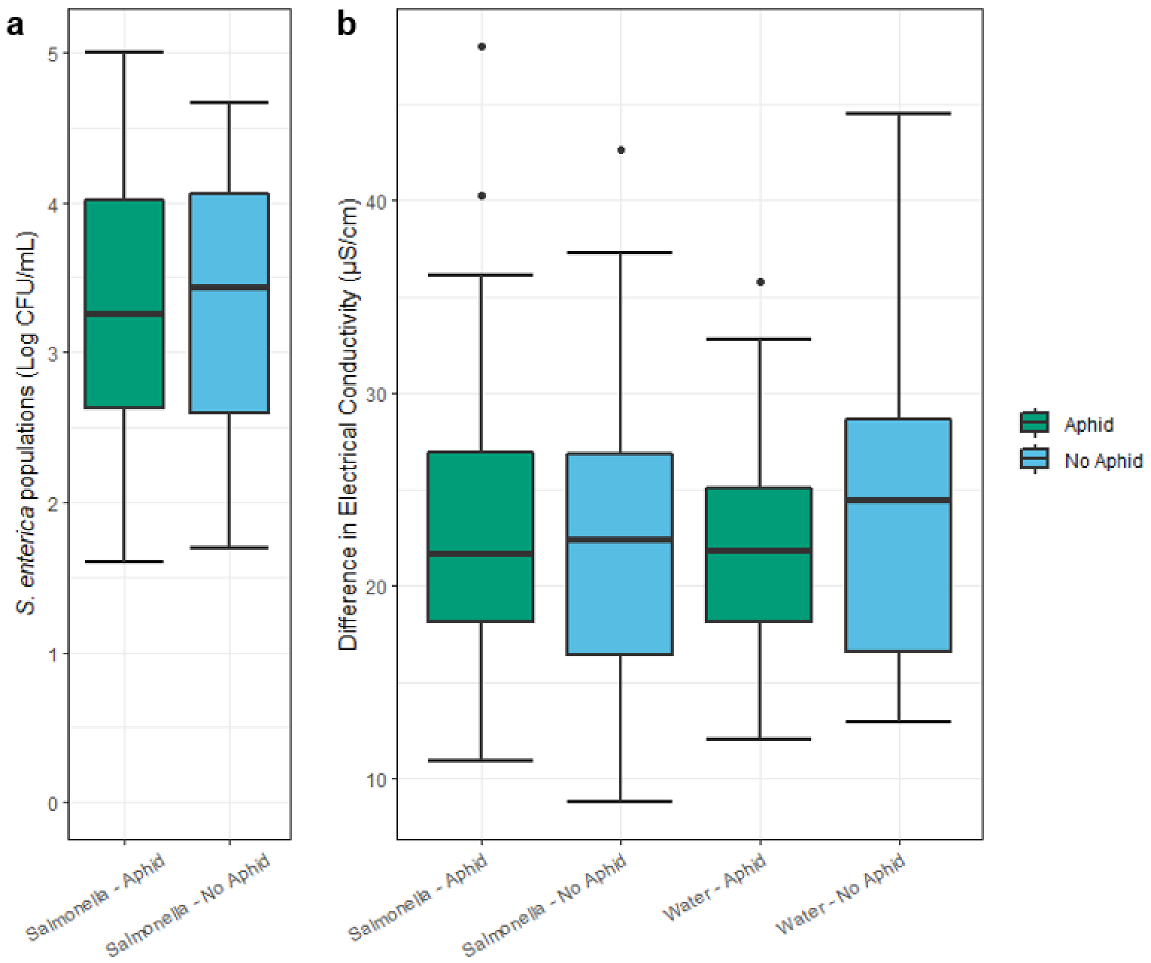

Supplemental Figure S7. *Myzus persicae* infestation on *S. enterica* inoculated tomato plants did not lead to heightened *bacterial* populations and electrical conductivity. Plants inoculated with either *S. enterica* or water were infested by apterous *M. persicae* or remained absent of insects. Empty clip cages were applied for treatment groups with no infestation. Electrical conductance was calculated by subtracting the final from the initial measurement for damaged and undamaged leaf discs and were used to evaluate the extent of electrolyte leakage over six hours. Each treatment group contains combined data from the tip, middle, and basal regions of leaves. *Salmonella enterica* populations were measured on water inoculated leaves but yielded 0 CFU and were thus excluded from the figure. Singular dots represent an outlier point.

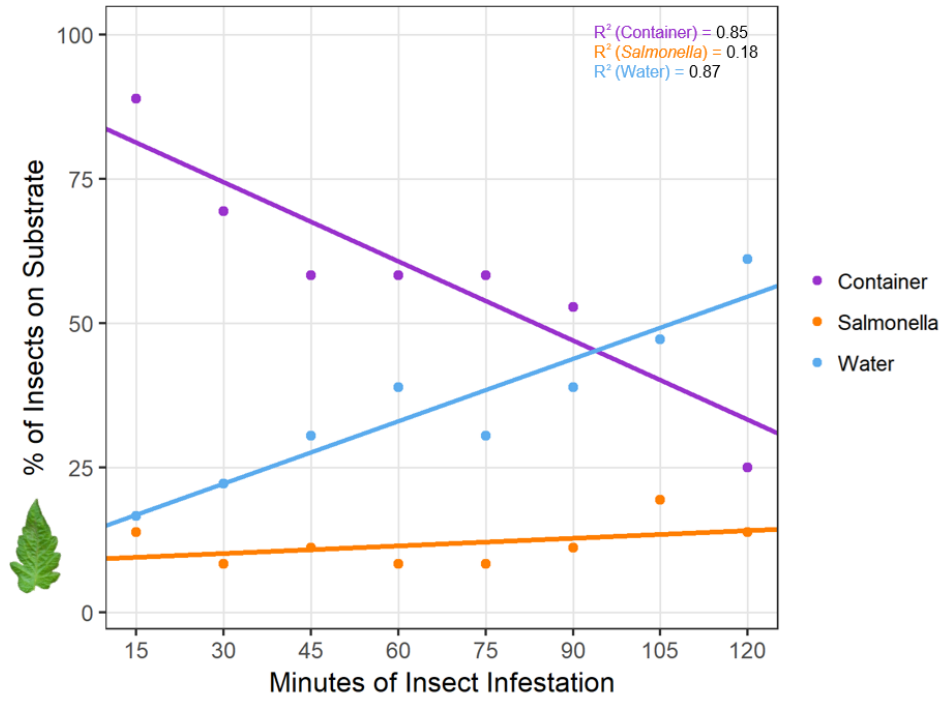

Supplemental Figure S8. The percentage of adult *M. quadrilineatus* resting on water inoculated tomato leaves increased over a two-hour duration. Over a two-hour period, replicate sets of adult *M. quadrilineatus* were placed in containers encasing a tomato leaflet still attached to the plant. One leaf had the distal (tip) half inoculated with *S. enterica*, and the basal portion remained inoculated with sterile water. Remaining leaves had inoculation positions switched. Observations were taken every 15 minutes, starting 15 minutes after the initial insect-plant exposure. The percent of insects on either substrate from two styles of inoculation (*S. enterica* and sterile water on the same leaf on either location) represent means of six experimental replicates and are represented as single points per 15 minute interval.

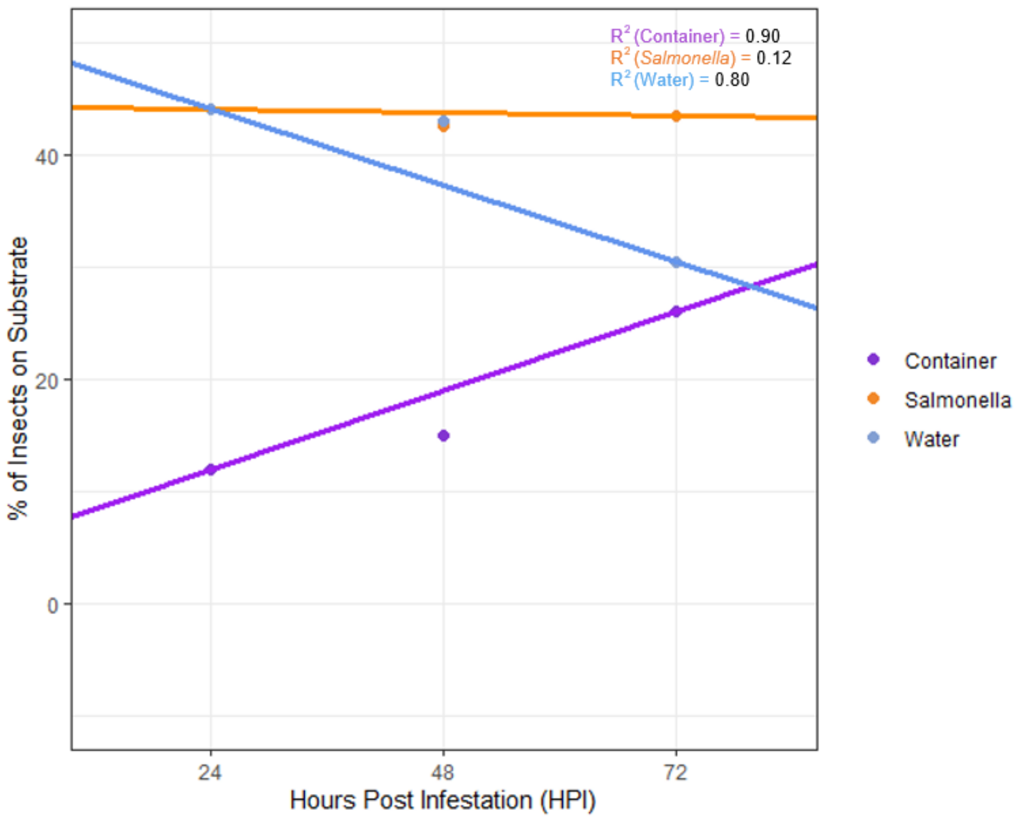

Supplemental Figure S9. The proportion of *M. persicae* that remain on leaflets inoculated with *S. enterica* over 72 hours. Tomato leaflets were partially inoculated with *S. enterica* on the right or left side of leaflets. One hour after *S. enterica* inoculation, one aphid was placed in a clip cage located on the middle of an inoculated tomato leaflet. Locations (Cage, *S. enterica* inoculated, or water inoculated regions) of individual insects were taken 24-, 48-, and 72 hours after the initial infestation period.

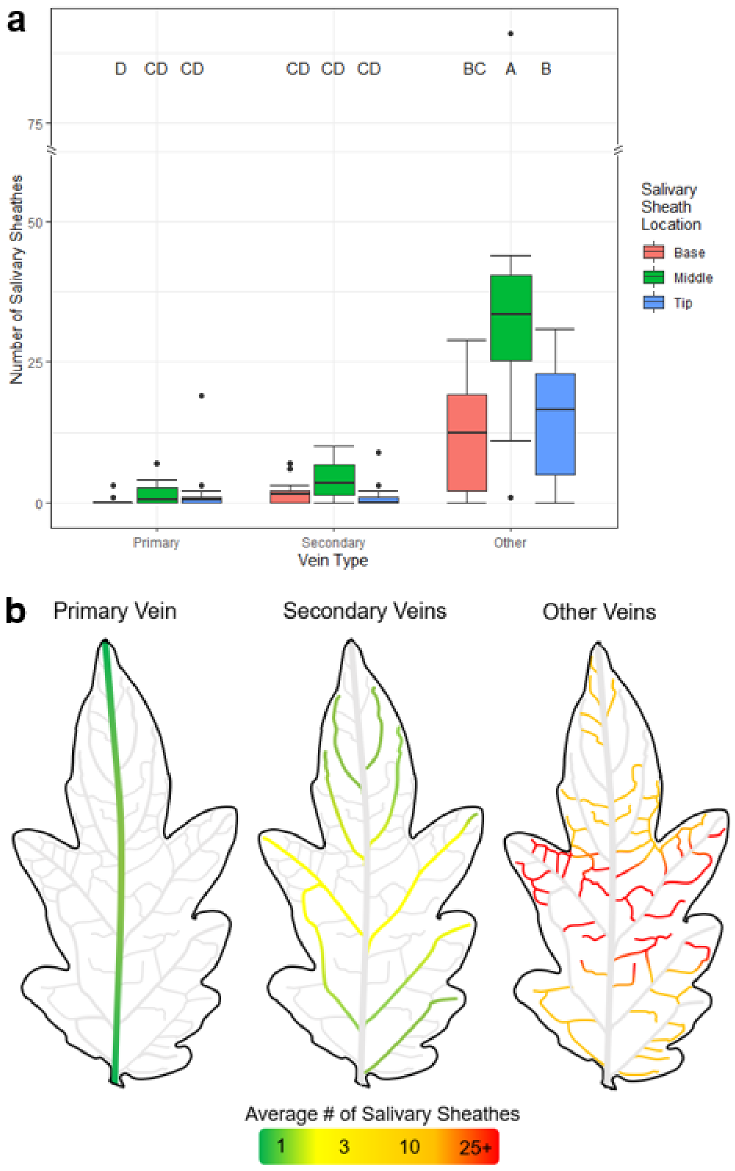

Supplemental Figure 10. *Macrosteles quadrilineatus* salivary sheathes are less frequently found on primary or secondary veins across water inoculated tomato leaflets. Five adult *M. quadrilineatus* were confined to a water inoculated tomato leaflet for 72 hours and were allowed to actively move and feed. Infested leaflets were removed and subjected to staining and clearing procedures. Letters above boxplots indicate significant differences between treatment groups within each experiment ($P < 0.05$), as detected by a one-way ANOVA (a). Salivary sheathes were assesed upon primary, secondary, and other lesser veins (b). Salivary sheathes from three experimental replicates (n = 15 leaflets) were combined and represented above.
